# Unveiling the Path to Resilience: Prioritizing Mental Health, Sleep, and Nutrition in the Post-COVID Era

**DOI:** 10.3390/healthcare11172463

**Published:** 2023-09-04

**Authors:** Susana Ramalho, Daniela Martins-Mendes, José Mário Macedo, Carla Barros, Carla Luis, Sara Sá, Álvaro Gestoso, Ana Cláudia Pereira, Pilar Baylina, Rúben Fernandes

**Affiliations:** 1 FP-I3ID, Instituto de Investigação, Inovação e Desenvolvimento, FP-BHS, Biomedical and Health Sciences, Universidade Fernando Pessoa, 4249-004 Porto, Portugal; srramalho.net@gmail.com (S.R.); danielamm@ufp.edu.pt (D.M.-M.); coelhomacedo@gmail.com (J.M.M.); cbarros@ufp.edu.pt (C.B.); carlaluis@med.up.pt (C.L.); saravsa@ufp.edu.pt (S.S.); agestoso@ufp.edu.pt (Á.G.); anacpereira@ufp.edu.pt (A.C.P.); 2 CECLIN, Centro de Estudos Clínicos, Hospital Fernando Pessoa, 4420-096 Gondomar, Portugal; 3 Faculdade de Ciências da Saúde, Universidade Fernando Pessoa, 4200-150 Porto, Portugal; 4 I3S, Instituto de Investigação e Inovação em Saúde, Universidade do Porto, 4200-135 Porto, Portugal; pilarbaylina@ess.ipp.pt; 5 Faculdade de Biologia, Universidade de Vigo, 36310 Vigo, Spain; 6 Faculdade de Medicina, Universidade do Porto, 4200-319 Porto, Portugal; 7 Faculdade de Ciências Humanas e Sociais, Universidade Fernando Pessoa, 4249-004 Porto, Portugal; 8 Escola S. Saúde, Instituto Politécnico do Porto, 4200-465 Porto, Portugal

**Keywords:** COVID-19 pandemic, mental health, resilience, sleep patterns, nutrition, obesity, well-being

## Abstract

The COVID-19 pandemic has disrupted daily life, impacting relationships, work, and education. This has led to increased stress, anxiety, and depression, along with altered sleep patterns and eating behaviors. Quarantine and isolation have worsened mental health, especially in children and the elderly, due to the loss of activities and physical contact. Sleep disorders and negative dreams perpetuate poor sleep quality, increasing the risk of health issues. Sedentary lifestyles and emotional effects contribute to unhealthy eating patterns and obesity, exacerbated by disrupted routines and limited outdoor activities. Addressing these challenges requires prioritizing mental health, promoting healthy sleep habits, and addressing obesity factors. The pandemic has profoundly affected human well-being, but resilience, mental health, sleep, and nutrition can enhance overall well-being and adaptability in the post-COVID era. This comprehensive opinion aims to raise awareness of the wide-ranging impacts of this pandemic on various aspects of human well-being and to emphasize the importance of implementing strategies that prioritize mental health, improve sleep habits, address eating behaviors, and foster resilience to navigate and thrive in the face of future challenges.

## 1. COVID-19, a Tsunami on Human Well-Being

The COVID-19 pandemic has had a profound impact on public health, resulting in significant changes across various dimensions of society. These changes have also influenced the way people perceive the world. The social implications of the pandemic include alterations in the daily lives of populations, which have had strong implications for interpersonal relationships within families, schools, and work environments. Furthermore, the pandemic has had effects on economics and geopolitics [1].

The consequences of the pandemic have had a significant impact on the health and economy of the global population, both directly and indirectly. Variations in lifestyle behaviors and patterns are supported by evidence. Apart from the visible effects of the virus itself, there are also invisible consequences on the mental health of the population [2]. The change in lifestyle, particularly during periods of confinement, has dramatically affected the physical and emotional health of individuals, leading to increased stress, anxiety, and depression. Changes in family dynamics, socio-professional habits, sleep patterns, and eating behaviors have also been observed [3].

COVID-19 has disrupted the normal human homeostasis by driving changes in metabolism, which are associated with respiratory problems, inflammation, and obesity [3]. Alterations in food patterns have also affected the brain–gut axis, ultimately impacting the metabolism of neurotransmitters and mental health [4]. The magnitude and speed of the pandemic’s impact on humanity have contributed to what has been described as a “tsunami of psychiatric illnesses”, leading to an imminent mental health crisis [5].

In the current scenario, daily life revolves around COVID-19, with a priority on physical and psychological well-being. The unknown nature of this illness has induced fear in people, making the need to communicate with doctors about their state a medical challenge [6]. However, the media can simultaneously serve as an excellent tool to foster confidence and adaptability, and minimize the negative repercussions of the pandemic and the resulting lifestyle changes [7,8]. Health organizations, epidemiologists, virologists, and public health specialists have provided relevant information and updated recommendations on the dissemination and lethality of COVID-19 [9].

Patients infected with the virus and their cohabitants experience physical and emotional discomfort due to the fear of severe illness and associated comorbidities [10,11]. They may also express concerns about discrimination and stigmatization from healthy individuals with whom they have lived or interacted [12]. This fear and anxiety can also be observed in individuals who do not have the disease, due to a lack of understanding about the illness [13,14,15]. Additionally, the precise effects of the virus on our health, including potential neuropsychiatric symptoms like cognitive decline, anxiety, depression, and anosmia, are known but still being studied, and the molecular mechanisms underlying the majority of these symptoms are still not fully understood [16].

It is expected that healthcare services will be equipped to address the mood disorders resulting from the pandemic and confinement. The circumstances for the delivery of primary and tertiary healthcare should be reassessed, with an emphasis on prioritizing individuals who display clinically severe difficulties and need proper management and follow-up [17]. In an ideal scenario, medical professionals would modify their protocols to provide quick, effective care while preserving mental health. Global healthcare systems must assist those who are suffering from mental illness and deal with the implications of the pandemic once it has passed [18]. The main impact of psychological disorders is on the quality of life, yet stigma continues to characterize these disorders, leading many individuals to experience shame and embarrassment [19]. The concerns of healthcare professionals and those on the frontline of the pandemic fight and its aftermath should not be overlooked, as they may require psychological help, necessitating a reevaluation of existing practices [18]. The need for support is further highlighted by the challenging and difficult experiences daily healthcare workers endure.

## 2. Post-Traumatic Stress Disorder during COVID-19 Pandemics

The SARS-CoV-2 epidemic has been linked to an increase in post-traumatic stress disorder (PTSD), stress, and anguish among patients, physicians, and other healthcare professionals, with an immediate and facing impact on mental health [20]. With vaccination as the only effective mitigation measure available, various measures were implemented to prevent the spread of the disease, including screening programs and social isolation. This led to a massive quarantine of millions of people, where telework became mandatory, classes shifted online, and only essential goods were allowed, creating uncertainty about the duration of confinement that negatively affected mental resilience [21]. While isolation is crucial for physical well-being, prolonged periods of isolation increase the risk of psychological disorders [10,11,13].

Studies have revealed that the outbreak has heightened psychological vulnerability and has become a significant concern for mental health [22]. The negative effects of quarantine have been greatly amplified, showing up as a range of psychopathological symptoms like melancholy, irritability, anxiety, fear, wrath, insomnia, and PTSD symptoms. These symptoms, in addition to social exclusion and confinement, increase the likelihood of developing PTSD and depressive disorders. Those who have had prior psychiatric disorders may experience worsened symptoms that could develop into PTSD or suicidal ideation [22,23]. These psychological challenges compromise well-being, result in changes in mental status, and may have significant neuropsychological effects. Long-term consequences for mental health include sadness, depression, anxiety, insomnia, denial, delirium, confusion, lack of attention and concentration, memory loss, and executive dysfunction [24,25].

In this complex scenario, both children and the elderly face mental health worries. Children miss out on educational, recreational, and peer possibilities, while older people are more susceptible to COVID-19 because of their weakened health. Although there are online platforms available, they cannot completely replace in-person contact, which is essential for well-being [26]. However, children at early ages and computer-illiterate elderly individuals face heightened anxiety and significant psychological stress factors, along with uncertainty about the future [14,26,27,28].

The deprivation of contact with family members and the broader social circle during isolation and/or hospitalization confirms the psychological instability experienced, resulting in high rates of post-traumatic symptoms [29]. Furthermore, COVID-19 has far-reaching implications for health-related quality of life, affecting various systems of the human body. Symptoms such as pain, fatigue, frailty, impaired ability to participate in family and community tasks, and altered self-identity and purpose are notable [30,31].

The psychosocial and mental health effects of the outbreak are particularly serious for vulnerable groups within the general population. Four groups are highlighted: (i) individuals directly or indirectly in contact with the virus; (ii) individuals vulnerable to biological and psychosocial stressors, including those affected by mental health disorders; (iii) health professionals exposed to higher risks; and (iv) individuals living with feelings of unpredictability and uncertainty, relying on various channels of social communication and the scientific community for news and information [17].

## 3. Sleep Disorders during COVID-19 Pandemics

Sleep disorders have emerged as a significant issue during the COVID-19 pandemic, affecting health and well-being [32]. The pandemic has brought about substantial changes in daily sleep habits, particularly among vulnerable groups, leading to alterations in sleep patterns and sleep quality. Individuals have reported an increase in the frequency of dreams and nightmares, with vivid memories of these dreams while awake. The content of these dreams tends to be predominantly negative, pessimistic, and hopeless, perpetuating negative beliefs and inadequate sleep hygiene habits, such as insomnia [33,34].

Sleep disorders emerge as a prominent focal point within the field of mental health concerns when connected with psychosocial stressors. Sleep is essential for both mental and physical well-being, and disturbances in the normal sleep cycle can result in insufficient rest; prolonged wakefulness, and an increased risk of insomnia, nightmares, excessive daytime sleepiness, and fatigue [34]. These sleep-related issues have become globally relevant during the pandemic, with studies indicating an increase in the number of individuals experiencing mental disorders and sleep disorders [35].

A recent review of the literature conducted in 2021 revealed an increased prevalence of sleep disorders in 2020, as highlighted by several studies conducted during the pandemic. These studies have associated changes in sleep patterns and quality with emotional effects stemming from isolation, quarantine, anxiety, stress, and financial losses [36]. Additional risk factors such as depression, anxiety, trauma, job insecurity, disruption of daily routines, increased technology use, exposure to conflicting and aggressive information from social media, illnesses, and loss of loved ones further exacerbate psychological health and contribute to sleep disturbances.

COVID-19 has had an impact on health and well-being that extends to sleep patterns, influencing people’s actions, daily lives, and worldview. Although poor sleep quality and insomnia symptoms have been documented, the particular content of pandemic-related dreams and nightmares remains mostly unknown. Consistent data regarding the association between poor sleep quality and insomnia symptoms are still lacking [28,37,38].

The relationship between sleep and health is bidirectional, with sleep being a fundamental aspect of human life. Maintaining good sleep hygiene is crucial for adopting a healthy lifestyle, enhancing overall health, and promoting resistance to infection when exposed to the virus. Conversely, sleep deprivation and the adoption of unhealthy sleep habits compromise well-being [39].

## 4. Sleep Disorders, Mood, and Human Health

Sleep disorders have wide-ranging effects on human health, extending beyond mood and mental well-being.

Mood disorders, such as depression, anxiety, and suicide attempts, had been long associated not only with sleep disturbances but also with influenza infection and infection with other coronaviruses (not SARS-CoV-2 virus) [40]. Inadequate sleep and sleep deprivation have been linked to a higher risk of infection, particularly in the context of the current COVID-19 pandemic [41]. Genomics has shown us that sleep disturbances may lead to a predisposition to viral infection, particularly viral lung diseases [42]. Thus, sleep deprivation, mood, and the increased risk for viral infection represent a vicious cycle [43]. Sleep-deprived individuals are not only more vulnerable to infections but also to other inflammatory conditions due to the negative impact on their immune system, such as chronic inflammatory state, autoimmune, neoplastic, neoplastic, and cardiometabolic diseases [41,44].

Also, the relationship between sleep and metabolic disorders (such diabetes and obesity) is understood. Sleep issues can cause stress hormones to be released, which can lead to obesity and insulin resistance. However, obesity can result in sleep disorders including sleep apnea. Hormones that regulate stress and hunger are disturbed by lack of sleep, which increases appetite and weight gain. This cycle emphasizes the importance of addressing both sleep and weight in order to break it [45,46].

Poor sleep disrupts hormones like leptin and ghrelin, affecting metabolism and hunger. This can boost appetite (ghrelin) and reduce feelings of fullness (leptin), promoting overeating and weight gain. Sleep issues release stress hormones like cortisol, causing increased blood pressure, insulin resistance, and abdominal fat accumulation. Impaired impulse control and decision making due to lack of sleep make healthy eating challenging [47,48]. Far from being completely understood, ghrelin and leptin have also been related to several mood disorders [49]. During the COVID-19 pandemic, the prevalence of metabolic comorbidities, such as dyslipidemia, hypertriglyceridemia, obesity, type 2 diabetes, and hypertension, has been observed to rise [50,51]. The compromised quality of sleep during COVID-19 contributes indirectly to these health concerns. The upcoming section also explores the direct connection between obesity and COVID-19.

The ability of the organism to repair and regenerate is closely tied to sleep. Yet, when sleep quality is compromised, this rejuvenating capacity is hindered, giving rise to potential health outcomes [52,53]. Addressing sleep disorders and promoting healthy sleep habits are imperative for safeguarding overall health. Adequate sleep enhances mood and the immune system, helps with weight management, and lowers the chance of acquiring a number of chronic disorders in addition to supporting mental health [39,44,45,47]. Prioritizing and maintaining good sleep hygiene should be an integral part of comprehensive health promotion efforts, particularly in times of crisis like the COVID-19 pandemic. By recognizing the significance of sleep hygiene in human health and taking proactive measures to address sleep disorders, individuals can enhance their overall well-being and resilience [44,50,51].

## 5. Behavior, Homeostasis, and Obesity during COVID-19 Pandemics

Obesity, a chronic, multifactorial, and complex condition, should be understood in the context of the issues it causes in a person’s life. Excessive body fat or adiposity not only compromises overall health but also increases the risk of long-term complications and reduces life expectancy [54]. Concerns about the increased risk of obesity and its associated morbidities, such as hypertension and diabetes mellitus, have been raised in relation to COVID-19 [55]. Evidence suggests that obesity may substantially enhance the likelihood of contracting COVID-19 infection and experiencing severe outcomes from the disease. Physiologically, obese individuals are prone to decreased airways and limited airflow, which can seriously affect their respiratory potential, particularly in the context of COVID-19 [56].

Social isolation measures implemented as a preventive measure during the pandemic have had negative effects on various aspects of human life. While effective in mitigating the spread of the disease, these practices have resulted in an increase in the incidence of mental health disorders [57,58]. The sedentary lifestyle imposed by the pandemic and the limitation of physical activity intensity have become prominent issues. The increased time spent sitting, sedentary behavior, fast-food intake, and prevalence of binge eating have been observed among individuals who are eutrophic, overweight, or obese [59,60]. The combination of emotional effects of COVID-19 and social isolation has the potential to lead to lasting consequences on metabolic health, exacerbating the prevalence of obesity and associated metabolic diseases [58].

Notably, children have also been affected by the pandemic, particularly concerning childhood obesity. The containment measures implemented have led to a deterioration in biopsychosocial health in children and adolescents, contributing to weight gain and its relationship with mental health determinants [61,62,63]. The restrictions on daily routines, closure of schools, cessation of organized physical activity, reduction in outdoor playtime, and deprivation of social contact have all played a role in the implementation of risk factors for obesity [64]. In addition, the increased use of information technologies as a means of compensating for the lack of interpersonal relationships has exacerbated sedentary behaviors and mental health risks, further contributing to weight gain [63,65].

Containment measures during pandemics have instigated significant transformations in familial routines. These changes have been associated with increased levels of stress, anxiety, and depression, accompanied by unhealthy eating patterns and sedentary behaviors. The prevalence, increase, or exacerbation of obesity is closely related to these factors [66]. The disruptions in daily routines, social support limitations, job loss, vulnerability to severe infection, and pre-existing mental health conditions have revealed a strong correlation between adverse mental health states and weight-related behaviors [67,68,69]. Stress from the development of sleep disorders is positively associated with obesity, hormone, and metabolic dysregulation through the hypothalamic–pituitary–adrenal axis [70,71]. The emotional states of loneliness, sadness, anguish, fear, and uncertainty also act as barriers to exercise and contribute to unhealthy eating habits [72,73].

During times of stress, individuals often resort to “emotional eating” as a coping mechanism, seeking comfort in high-calorie, high-fat, and sugar-rich foods. The consumption of these foods stimulates the brain’s reward centers, offering a temporary respite from stress and negative emotions [74,75,76]. However, the repeated engagement in stress-induced eating can lead to alterations in brain function, fostering increasingly compulsive behaviors and a preference for indulgent foods during challenging events, such as the COVID-19 pandemic [77,78]. It is important to note, though, that the effects of “comfort food” are transitory, and the notion of their long-lasting benefits is a widespread misconception [77,79].

Research has demonstrated the significant influence of psychological factors on eating behaviors during the pandemic. The experience of financial hardship has been associated with an increased likelihood of engaging in health risk behaviors, including emotional eating [79]. Moreover, the implementation of health safety practices, such as social distancing and stay-at-home orders, has impacted individuals’ grocery shopping behaviors, potentially influencing the availability and choice of comfort foods [66]. Consequently, the pandemic has witnessed shifts in food purchasing and eating behavior, with individuals exhibiting changes in their consumption patterns, preferences, and reliance on specific food types [62,80].

Understanding the dynamics of emotional eating during times of stress, such as the ongoing COVID-19 pandemic, is crucial for promoting healthier coping mechanisms and mitigating the potential negative consequences on individuals’ well-being [78,81]. By addressing the underlying psychological factors and providing support for healthier alternatives, individuals can develop more sustainable strategies to manage stress and maintain a balanced approach to their dietary choices [80,81,82].

## 6. The Path to Resilience in the Post-COVID Pandemic Era

The COVID-19 pandemic has had significant implications for individuals’ emotional well-being, physical health, and eating habits. The disruption of daily routines, social isolation, increased sedentary behavior, and stress have contributed to an increase in obesity and its associated health problems [80,81]. Understanding and addressing the complex interplay between mental health, physical activity, eating habits, and obesity are crucial in mitigating the long-term consequences of the pandemic on individuals’ overall well-being [73,81].

The path toward resilience in the context of the COVID-19 pandemic involves a multifaceted approach that encompasses various aspects of physical, mental, and social well-being. Here are some key components that contribute to building resilience: mental health support and awareness; promoting healthy sleep habits; enhancing physical activity and healthy nutrition habits; supporting continuous education, literacy, and awareness; promoting coping mechanisms; and social and community support [83]. Prioritizing mental healthcare and resources in the aftermath of COVID-19 is critical. Predictors of post-pandemic suffering and resilience have been discovered, emphasizing the significance of hope and adaptive coping mechanisms. Furthermore, assisting vulnerable populations, such as industrial employees, and promoting communal solidarity are critical for long-term mental health. The resilience of Latinx youth to COVID-19 stress stresses the need of family support and appropriate coping methods [84,85,86].

In terms of diet and nutrition, evidence suggests that some nutritious nutrients enhance the body’s defenses against viral infection. The human body is better able to fight viruses when the diet is well balanced. A healthy immune system depends on eating a variety of foods that are high in vitamins, minerals, and antioxidants. Our immune system benefits greatly from vitamins C, D, zinc, and selenium, among other nutrients. These crucial nutrients are provided to us by foods including fruits, vegetables, whole grains, lean proteins, and healthy fats. Inflammatory foods can damage our health and immune systems. Consuming meals like fatty fish, olive oil, nuts, seeds, and colorful fruits and vegetables can minimize inflammation. Our immune system is impacted by the condition of our gut. Fermented foods like yogurt, kefir, sauerkraut, and kimchi are beneficial for our digestive systems and general health. Dietary fiber from grains, beans, fruits, and vegetables also benefits our digestive system.

A well-balanced diet rich in vitamins, minerals, antioxidants, and other nutrients is crucial for supporting a strong immune system. Nutrients like vitamin C, vitamin D, zinc, and selenium play specific roles in immune function [87]. A diverse range of fruits, vegetables, whole grains, lean proteins, and healthy fats can provide the necessary nutrients for optimal immune responses. Chronic inflammation can weaken the immune system and contribute to various health issues. A diet rich in anti-inflammatory foods, such as fatty fish (like salmon and mackerel), olive oil, nuts, seeds, and colorful fruits and vegetables, can help mitigate inflammation and promote overall health [88]. The gut microbiome plays a crucial role in immune function and overall well-being [89]. Fermented foods like yogurt, kefir, sauerkraut, and kimchi are also beneficial for gut health and improves eubiosis (good microbiota balance) [90,91]. A diet high in fiber from whole grains, legumes, fruits, and vegetables can support a healthy gut microbiome.

In sum, a healthy diet and lifestyle may protect against adverse mental health outcomes, which is especially crucial during stressful times, such as the COVID-19 pandemic.

## 7. Conclusions

In conclusion, the COVID-19 pandemic has had far-reaching effects on human well-being, creating a tsunami of challenges across various aspects of society. The social implications of the pandemic have resulted in significant changes in daily life, impacting interpersonal relationships, work environments, and education. The pandemic has also caused both direct and indirect consequences on physical and mental health, leading to increased stress, anxiety, and depression. Lifestyle changes, such as altered sleep patterns and eating behaviors, have further contributed to the disruption of human homeostasis and metabolic health.

The pandemic triggered a mental health crisis, escalating PTSD, stress, and distress. Quarantine and isolation worsened vulnerability, fostering psychopathological symptoms. Children and the elderly suffered from the loss of education, social activities, and contact. Isolation led to heightened post-traumatic symptoms. Sleep disorders surged, affecting patterns and quality. Dreams, often negative, reinforced pessimistic beliefs and poor sleep hygiene. Coupled with stress, sleep disorders raised risks of physical and mental issues. Poor sleep is linked to depression, anxiety, impaired cognition, and reduced well-being.

Furthermore, the pandemic has had implications for obesity and eating behaviors. The sedentary lifestyle imposed by social distancing measures and the emotional effects of the pandemic have contributed to unhealthy eating patterns, stress-induced emotional eating, and weight gain. The changes in daily routines, closure of schools, and limited outdoor activities have also impacted childhood obesity rates.

Addressing these challenges requires a comprehensive approach. Healthcare services should prioritize mental health support and provide appropriate management and follow-up for individuals experiencing clinically significant problems. Efforts should be made to reduce the stigma surrounding mental health disorders and ensure that healthcare professionals themselves receive the necessary psychological support. Additionally, promoting healthy sleep habits and addressing sleep disorders should be part of comprehensive health promotion efforts. This includes recognizing the bidirectional relationship between sleep and overall health and implementing strategies to improve sleep quality and hygiene. Finally, addressing the issues of obesity and unhealthy eating behaviors requires a focus on promoting healthier coping mechanisms, providing access to nutritious food options, and fostering a supportive environment for individuals to make positive lifestyle changes.

Further investigation is needed to understand the links between the COVID-19 pandemic, emotional well-being, sleep problems, and obesity. Investigating the long-term effects of pandemic-induced stress, disruptions in daily habits, and emotional eating on mental health and weight management could yield useful results. Research might also concentrate on creating effective interventions to improve healthy coping processes, reduce emotional eating, and boost physical activity. Understanding the complex relationship between sleep quality, metabolic health, and obesity during and after a pandemic could also assist and guide initiatives to promote overall well-being. Investigating these knowledge gaps can lead to practical solutions for increasing resilience and reducing the negative effects of the pandemic on mental and physical health.

Overall, the COVID-19 pandemic has had a profound impact on human well-being, affecting physical and mental health, sleep patterns, and eating behaviors. Addressing these challenges requires a holistic approach that prioritizes mental health support, promotes healthy sleep habits, and addresses the underlying factors contributing to obesity and unhealthy eating behaviors. By taking proactive measures to mitigate the negative effects of the pandemic, individuals can enhance their overall well-being and resilience in the face of future challenges.

## Data Availability

Not applicable.
